# Prevalence and Correlates of Cognitive Impairment in Kidney Transplant Patients Using the DemTect—Results of a KTx360 Substudy

**DOI:** 10.3389/fpsyt.2019.00791

**Published:** 2019-10-31

**Authors:** Mariel Nöhre, Maximilian Bauer-Hohmann, Felix Klewitz, Eva-Marie Kyaw Tha Tun, Uwe Tegtbur, Lars Pape, Lena Schiffer, Martina de Zwaan, Mario Schiffer

**Affiliations:** ^1^Department of Psychosomatic Medicine and Psychotherapy, Hannover Medical School, Hannover, Germany; ^2^Project Kidney Transplantation 360° (NTX360°), Hannover Medical School, Hannover, Germany; ^3^Department of Psychosomatic Medicine and Psychotherapy, University of Göttingen Medical Centre, Göttingen, Germany; ^4^Department of Sports Medicine, Hannover Medical School, Hannover, Germany; ^5^Department of Pediatric Kidney, Liver, and Metabolic Diseases, Hannover Medical School, Hannover, Germany; ^6^Department of Nephrology and Hypertension, Hannover Medical School, Hannover, Germany; ^7^Department of Nephrology and Hypertension, University Hospital Erlangen, Erlangen, Germany

**Keywords:** cognitive functioning, cognitive impairment, kidney transplantation, renal transplantation, DemTect

## Abstract

Cognitive impairment in kidney transplantation (KTx) patients is associated with allograft survival and mortality. However, the prevalence of cognitive impairment after KTx is still understudied. Thus, we aimed to assess the prevalence of cognitive impairment in KTx patients and to identify sociodemographic, medical, donation-specific, and psychological variables associated with cognitive impairment. In this cross-sectional two-center study, 583 KTx patients participated in a structured post-transplant care program. The DemTect was used to assess cognition, and cognitive impairment was defined as a score of < 13. Mean age was 52.11 years, 59% were male, 27.4% had ≥12 years of school attendance, and 85.9% had hypertension. The prevalence of cognitive impairment was 15.6%. Cognitive impairment was significantly associated with higher age, male sex, lower educational level, subjective perception of cognitive decline, higher rates of hypertension, lower kidney functioning, and obesity (BMI > 30 kg/m^2^). Using logistic regression analysis, all variables except age remained significant. Our results suggest that cognitive impairment affects a significant number of patients after KTx. Transplant centers may consider screening for cognitive impairment using objective tests, especially in patients with a high-risk profile. Furthermore, studies with longitudinal designs are required in order to assess moderators and mediators for cognitive trajectories.

## Introduction

Kidney transplantation (KTx) is the treatment of choice for patients with end-stage renal disease (ESRD) (1). Due to low numbers of deceased organ donors and an increase of ESRD patients, in general, the current waiting time of KTx candidates who undergo dialysis therapy is about 6 to 7 years in Germany (2, 3). It is known that dialysis therapy is inferior to KTx regarding quality of life, morbidity, and mortality of ESRD patients.

Previous studies have shown that cognitive impairment is a significant problem for patients on dialysis with prevalence rates ranging between 50% and 87% (4, 5). Additionally, cognitive impairment correlated with a higher risk of hospitalization and morbidity (6). Several risk factors for cognitive impairment in patients on dialysis such as reduced creatinine clearance, elevated homocysteine levels, arteriosclerosis, and rapid fluid and osmotic shifts have been identified so far (7, 8). In a recent meta-analysis, Joshee et al. (7) found moderate to substantial improvements in several cognitive domains based on neuropsychological tests in patients after KTx compared to their pre-donation scores. At the same time, patients after KTx still performed significantly lower in some domains compared to healthy controls (7). Thus, cognitive impairment may not be entirely reversible and cognitive functioning might deteriorate long term due to comorbid medical conditions and the neurotoxicity of immunosuppressive medication, especially calcineurin inhibitors (CNI) (7). However, no information on the prevalence of cognitive impairment after KTx is provided in this study. To our knowledge, the prevalence of cognitive impairment in KTx patients was explicitly evaluated in one study so far (9). Gupta et al. found a prevalence of cognitive impairment of 58.0% in 226 KTx patients by using the Montreal Cognitive Assessment (MoCA) (9, 10).

Additionally, two recent studies are evaluating cognitive impairment in KTx patients in two at least partially overlapping samples using the Modified Mini-Mental State Examination (3MS) (11, 12). The baseline prevalence rates of cognitive impairment were assessed immediately before transplantation and were 7.2% (11) and 10.0% (12), respectively. After transplantation, the authors described an improvement of cognitive functioning; however, prevalence rates of cognitive impairment after transplantation were not reported.

While neuropsychological tests provide a detailed evaluation of the participants’ cognitive performance, they are time-consuming and cost-intensive and therefore not suitable for the use in daily clinical practice. Also, patients, especially those already experiencing cognitive impairment, might not agree to participate in detailed neuropsychological testing. Thus, we decided to use the DemTect which is suitable for the use in daily clinical practice and is a sensitive screening instrument to assess also mild cognitive impairment (MCI) (13). MCI is defined as the state between cognitive changes of aging and early dementia and is characterized by a decline of cognitive functioning compared to the past, but typically does not severely affect daily life (14, 15). The DemTect can detect MCI with a sensitivity of 80% and shows similar accuracy compared to the CERAD (Consortium to Establish a Registry for Alzheimer’s disease) test battery (16–18).

Additionally, the DemTect provides age-adapted scoring routines and is not education-dependent (19). Above that, there is an equivalent test for the DemTect-A available (DemTect-B). The versions can be mutually interchanged and, therefore, are helpful for follow-up investigations. As far as we know, the DemTect is the only instrument available in German which has a parallel version (20). These characteristics distinguish the DemTect from other screening instruments and make it an appropriate tool for our purpose.

Since cognitive impairment may interfere with the necessary self-care on the part of the transplant patients and has shown to be associated with inferior medical outcomes (12) screening for cognitive impairment after KTx is crucial. Reliable data on cognitive impairment in well-defined cohorts of KTx patients are scarce. Thus, the aim of this study was (1) to investigate the prevalence of cognitive impairment in KTx patients using the DemTect; (2) to identify sociodemographic, medical, donation-specific, and psychological variables associated with cognitive impairment; and (3) to compare our findings with previous research.

## Methods

### Participants

Participants were recruited within the structured post-transplant care program (KTx360°) conducted at the transplant centers of Hannover Medical School and Hannoversch Münden in Lower Saxony, Germany (21). Within this ongoing study, 1,200 KTx patients of at least 16 years of age were approached between May 2017 and December 2018. Six hundred nineteen (51.6%) KTx patients took part in the study. The non-participants indicated that they were not interested in participating in a structured follow-up program. For the non-participants, only basic information was available. They were significantly older, had a longer time since KTx, had more often received an organ from a living kidney donor, had a higher rate of diabetes, and a lower rate of anemia compared to the participants. There was no significant difference regarding sex, renal functioning, and hypertension.

A medical doctor or psychologist performed a psychosocial risk assessment in all participating KTx patients. Parts of the assessment were a structured interview to detect mental comorbidity as well as an evaluation of adherence behavior. Above that, the assessment included the DemTect as a screening for cognitive impairment (13). Thirty-six of the 619 patients did not complete the DemTect due to the inability to speak fluent German, hearing or visual impairment or a known history of severe developmental delay. Five hundred eighty-three patients completed the DemTect and were included in the analyses.

The study was approved by the institutional ethics review board of Hannover Medical School (number 3464-2017), and all participants gave written informed consent.

### Assessment Instruments

#### Cognitive Functioning

Cognition was assessed with the DemTect, a brief and sensitive screening instrument for mild cognitive impairment (13). It can be performed in 8 to 10 min and consists of five tasks: a word list, a number transcoding task, a word fluency task, digit span reverse, and delayed recall of the word list. A summary of the different cognitive abilities evaluated in the five subtests can be found in Table 1. A maximum score of 18 can be reached. A score of 13 to 18 corresponds to an age-adequate cognitive performance, a score of 9 to 12 corresponds to mild cognitive impairment, and a score of 8 or below raises the suspicion of the presence of dementia. To control for age effects, there are different cut-offs for score transformation for participants younger than 60 years, and for those who are 60 years or older.

There are calculated bidirectional conversion tables available allowing the conversion and comparison of the German versions of the DemTect and the two other frequently used screening instruments MoCA and Mini-Mental State Examination (MMSE) (10, 22, 23).

**Table 1:** Cognitive domains evaluated with the subtests of the DemTect [adapted from Kessler et al. (20)].

Subtests	Cognitive abilities
Wordlist	Verbal memory
Number transcoding	Lexical processing, syntactic processing, language processing (reading and writing), executive functioning
Semantic word fluency test	Attention, working memory, cognitive flexibility, problem-solving, imagery, semantic memory, language, speed of processing
Digit span reverse	Working memory
Wordlist delayed recall	Verbal long term memory

Additionally, patients were asked if they have experienced subjective changes in their cognitive functioning since transplantation.

#### Symptoms of Depression and Anxiety

Anxiety and depression were measured with the German version of the Hospital Anxiety and Depression Scale (HADS) (24, 25). The self-report instrument is specifically designed to assess levels of anxiety and depression in physically ill patients. There are two subscales “depression” and “anxiety.” Each of the two scales consists of seven items. Each item is scored from 0 to 3, yielding a total score between 0 and 21. Higher scores indicate higher levels of depression or anxiety. Scores ≥ 11 are indicative of clinically relevant symptoms of anxiety or depression. Cronbach’s α in our sample was 0.872 for depression and 0.827 for anxiety.

#### Adherence

The Medication Adherence Rating Scale (MARS-D) is a five-item self-report instrument assessing non-adherent behavior on a five-point Likert scale (26). In our study, the German version of the questionnaire was used (27). The total score ranges from 5 to 25, with higher scores indicating higher adherence. Patients were considered to be non-adherent if they scored below 25 (28, 29). For our study, patients were asked only to consider immunosuppressive medication in their ratings. The MARS-D was adapted accordingly with the approval of the original authors. Cronbach’s α in our sample was 0.669.

#### Medical Parameters

The medical parameters estimated glomerular filtration rate (eGFR) and creatinine at the time of administration of the DemTect and the presence of hypertension, renal anemia, and diabetes mellitus were taken from the medical records. Bodyweight and body height were measured using a standardized scale. Patients with a body mass index (BMI) of 30 kg/m^2^ or above were rated as obese.

#### Sociodemographic and Donation-Specific Variables

Sociodemographic and donation-specific variables including sex, age, partnership status, level of education, donation type, time since KTx, dialysis treatment, and dialysis duration were assessed using a self-report questionnaire, and missing information were obtained from the medical records.

#### Statistics

Questionnaire scores were calculated for the entire sample and separately for patients with and without cognitive impairment. For each variable descriptive statistics (percentage or mean and standard deviation) were calculated.

We used Shapiro-Wilk test and Kolmogorov-Smirnov test to evaluate data distribution. As our data were not normally distributed, Mann-Whitney U tests were used for comparison of continuous data between the sample with cognitive impairment and the sample without cognitive impairment. Chi-square tests were used for categorical data.

Binary logistic regression analysis with the DemTect (dichotomous) as the dependent variable and variables that were significant in the univariate tests as the independent variable were conducted. All patients with complete data sets (n = 552) were included in this analysis.

For all analyses, *P* < 0.05 was considered statistically significant. All statistical analyses were performed using IBM^®^ Statistical Software Package of Social Science (SPSS^®^, Chicago, IL, USA) version 25.

## Results

### Participant Characteristics

Participant characteristics are summarized in Table 2. Our sample comprised 239 women (41%) and 344 men (59%). The mean age at the time of assessment was 52.11 years (SD 14.25). 27.4% reported a school attendance of 12 years or more. 29.9% of the participants had received their kidney from a living kidney donor.

**Table 2:** Comparison of demographic and clinical characteristics between KTx patients with and without cognitive impairment.

Patient characteristics	All N = 583 (100%)^a^	DemTect <13 N = 91 (15.6%)^b^	DemTect ≥ 13 N = 492	Statistics χ^2^ test, Mann-Whitney U test
**Age (years), mean (SD)**	52.11 (14.25)	55.32 (13.69)	51.46 (14.23)	***Z*** ** =** −**2.254, ** ***P*** ** = 0.024**
**Female gender**	239 (41%)	25 (27.5%)	214 (43.5%)	**χ** **^2^** ** = 8.151 (** ***df*** ** = 1), ** ***P*** ** = 0.004**
**≥12 years school attendance (n = 552)**	151 (27.4%)	12 (14.5%)	139 (29.6%)	**χ** **^2^** ** = 8.177 (** ***df*** ** = 1),** *** P*** ** = 0.004**
**Hypertension**	501 (85.9%)	86 (94.5%)	415 (84.3%)	**χ** **^2^** ** = 6.553 (** ***df*** ** = 1), ** ***P *** **= 0.010**
**Renal anemia**	165 (28.3%)	22 (13.3%)	143 (86.7%)	χ^2^ = .905 (*df* = 1), *P* = 0.342
**Diabetes mellitus (1,2, and NODAT)**	101 (17.4%)	20 (24.2%)	81 (16.5%)	χ^2^ = 1.608 (*df* = 1), *P* = 0.205
**eGFR (ml/min/1.73 m** **^2^** **), mean (SD)**	45.75 (18.44)	40.46 (19.05)	46.73 (18.18)	***Z*** ** = −3.553, ** ***P*** ** = 0.001**
**Creatinine (mg/dl), mean (SD)**	1.77 (0.69)	2.04 (0.79)	1.73 (0.67)	***Z*** ** = −3.884, ** ***P*** ** = 0.001**
**eGFR < 30 ml/min/1.73 m** **^2^**	120 (20.6%)	29 (31.9%)	91 (18.5%)	**χ** **^2^** ** = 8.401 (** ***df*** ** = 1), ** ***P*** ** = 0.004**
**Living donation**	185 (29.9%)	20 (22.0%)	157 (31.9%)	χ^2^ = 3.583 (*df* = 1), *P* = 0.058
**Time since KT (months), mean (SD)**	66.06 (68.58)	69.00 (76.4)	65.5 (67.11)	*Z* = −0.161, *P* = 0.872
**Time on dialysis (months), mean (SD)**	60.25 (49.60)	62.48 (44.33)	59.84 (50.54	*Z* = −0.815, *P* = 0.415
**MARS-D score (adherence), mean (SD)**	24.35 (1.33)	24.34 (0.96)	24.35 (1.38)	*Z* = −0.665, *P* = 0.506
**HADS Anxiety score, mean (SD)**	5.13 (3.92)	5.18 (4.29)	5.13 (3.86)	*Z* = −0.255, *P* = 0.799
**HADS Depression score, mean (SD)**	4.35 (4.00)	4.85 (4.69)	4.25 (3.87)	*Z* = −0.675, *P* = 0.500
**Subjective change of cognitive functioning (n = 574)**				
** Worse**	133 (23.2%)	26 (28.9%)	107 (22.1%)	**χ** **^2^** ** = 6.889 (** ***df*** ** = 2), P = 0.032**
** Improved**	61 (10.6%)	3 (3.3%)	58 (12.0%)	****
** No change**	380 (66.2%)	61 (67.8%)	319 (65.9%)	****
**BMI ≥30 kg/m** **^2^** ** (n = 425)**	75 (17.6%)	19 (29.7%)	56 (15.5%)	**χ** **^2^** ** = 7.516 (** ***df*** ** = 1), P = 0.006**

^a^Percent of all participants, ^b^percent of the demographic variable or clinical characteristic.

P < 0.05 was considered statistically significant, DemTect <13 = patients with cognitive impairment, DemTect ≥13 = patients without cognitive impairment; statistically significant results (P < 0.05) are shown in boldface. The significance of bolded texts is p < 0.05 as suggested.

Regarding medical conditions, 85.9% were diagnosed with hypertension, 28.3% with renal anemia, and 17.4% with diabetes mellitus (type 1, type 2 or new-onset diabetes after transplantation (NODAT)). 20.6% had an eGFR below 30 ml/min/1.73 m^2^, corresponding to a severe reduction of the glomerular filtration rate (30). The BMI was available for 425 patients. Of those, 17.6% were obese.

### Prevalence of Cognitive Impairment

**Figure 1:**
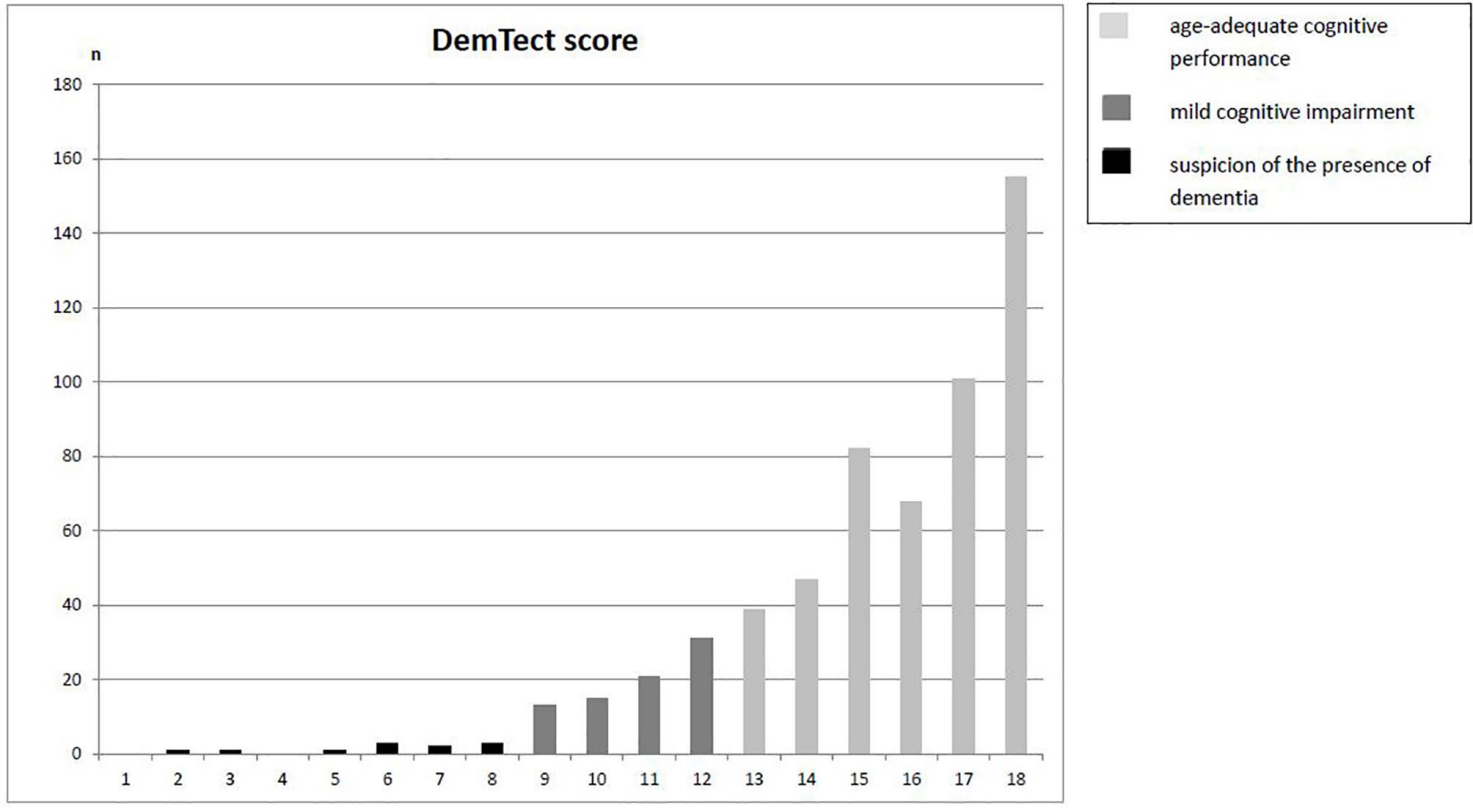
Distribution of DemTect sum scores.

**Figure 2:**
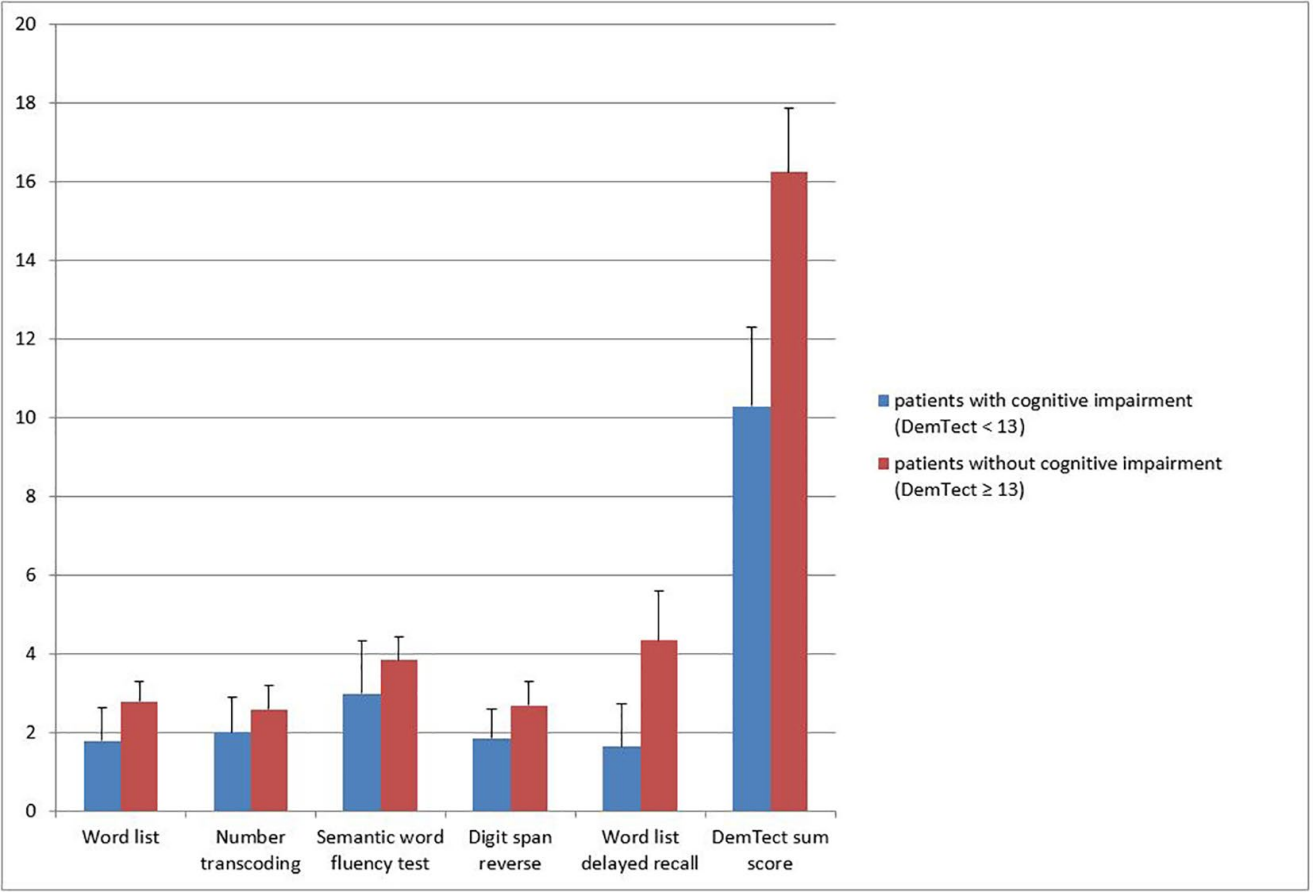
Mean subtest scores and mean sum score for patients with and without cognitive impairment.

In the screening for cognitive impairment using the DemTect, 492 participants (84.4%) showed normal cognitive functioning (DemTect ≥ 13), 80 participants (13.7%) reached a DemTect score between 12 to 9 corresponding to mild cognitive impairment, and 11 participants (1.9%) had a DemTect score of 8 or below, indicative for severe cognitive impairment. Mild and severe impairments were taken together for further analyses (n = 91, 15.6%) (**Figure 1**). **Figure 2** shows the mean score for each subtest as well as the mean sum score for patients with and without cognitive impairment. Using Mann-Whitney U tests we found a statistically significant difference for each subtest and sum score.

To allow comparison with the study of Gupta et al. (9) the DemTect scores were converted into MoCA scores, leading to a mean value of 27.16 (SD 3.18) with 22.3% of the patients reaching a score below 26, indicating cognitive impairment.

When asked about subjective changes in cognitive functioning after KTx, answers were available from 574 patients. There were 23.2% reported worsened cognitive functioning, while 10.6% reported improved cognitive functioning, and 66.2% did not perceive any change in cognitive functioning.

### Comparison of Demographic and Clinical Characteristics Between KTx Patients With and Without Cognitive Impairment

KTx patients with cognitive impairment (age-adjusted score < 13) differed significantly from those without cognitive impairment (age-adjusted score ≥13) in all five subtests (data not shown). KTx patients with cognitive impairment were significantly older, the percentage of male patients was significantly higher, and the rate of patients with ≥12 years of education was significantly lower compared to those without cognitive impairment (**Table 2**).

In the group with cognitive impairment, significantly more patients reported subjective worsening of cognitive functioning compared to the other group (**Table 2**).

The percentage of patients with hypertension was significantly higher in the group with cognitive impairment. No differences between the groups were found regarding the rate of renal anemia and diabetes mellitus. However, KTx patients with cognitive impairment had significantly lower eGFR, and higher creatinine values, and the percentage of patients with an eGFR below 30 ml/min/1.73 m^2^ was significantly higher in the group with cognitive impairment compared to the group without cognitive impairment (**Table 2**).

There were no differences regarding donation type, time since KTx, and time on dialysis between patients with and without cognitive impairment. Additionally, the groups did not differ regarding adherence rates measured with the MARS-D and symptoms of depression and anxiety evaluated with the HADS (**Table 2**).

In the subgroup of 425 patients with measured weight and height data, significantly more patients with cognitive impairment were obese at the time of the assessment compared to patients without cognitive impairment (**Table 2**).

Binary logistic regression analysis with the DemTect (dichotomous) as the dependent variable and sex, age, educational level, the presence of hypertension, and eGFR as independent variables was performed. The results are reported in **Table 3**. All independent variables except age remained significant.

**Table 3:** Binary logistic regression analysis with DemTect (dichotomous) as the dependent variable and variables that were significant in the univariate tests as the independent variable (n = 552).

Variable	Regression coefficient B	Standard error	Wald	df	Sig.	Exp(B) OR	95% CI, lower	95% CI, upper
Presence of hypertension	0.972	0.488	3.962	1	0.047	2.643	1.015	6.881
Male gender	0.712	0.267	7.118	1	0.008	2.037	1.208	3.436
<12 years of school attendance	0.892	0.336	7.051	1	0.008	2.441	1.263	4.716
Age (years)	0.009	0.009	0.937	1	0.333	1.009	0.991	1.028
eGFR (ml/min/1.73 m^2^)	−0.020	0.008	7.014	1	0.008	0.980	0.965	0.995
Constant	−3.376	0.841	16.114	1	0.000	0.034		

Goodness of fit for the final model was based on Hosmer-Lemeshow test which was not significant (χ^2^ = 3.273, df = 8, P = 0.916).

## Discussion

The primary aim of this study was to assess the prevalence and correlates of cognitive impairment in KTx patients using the DemTect. This is of clinical interest, since recent studies suggest that cognitive impairment might be associated with an increased risk of all-cause graft loss in KTx patients (12). In our study, 15.6% of the participants had DemTect scores below 13, indicating cognitive impairment. These numbers are significantly lower than the results of Gupta et al. (9), who found cognitive impairment in 58% of the KTx patients in their cohort, and more in line with the results of Chu et al. and Thomas et al. (11, 12) who reported prevalence rates of 7.2% and 10%, respectively, in patients immediately before KTx using a modified version of the MMSE (3MS). Since the studies used different tests to evaluate cognitive impairment, the results are not directly comparable. Thus, we transformed the DemTect scores into MoCA scores using suggested conversion tables (23). However, the prevalence rate of cognitive impairment stayed significant lower in our sample (22.3%) compared to the rate reported by Gupta et al. (9). Unfortunately, no transformation scores into the 3MS are available. While the participants in the Gupta et al. (9) study had higher educational levels (57.5% ≥ 12 years vs. 27.4% ≥ 12 years) and better renal functioning (eGFR 52 ml/min/1.73 m^2^ vs. 45.75 ml/min/1.73 m^2^), they also had a markedly higher obesity rate (50.9% BMI > 30 kg/m^2^ vs. 17.6% BMI > 30 kg/m^2^) compared to the sample in our study which might at least in part account for this difference.

The DemTect results were significantly associated with the subjective experience of change of cognitive functioning since transplantation. However, most participants reported no subjective change in cognitive functioning (66.2%), and more participants reported a decline (23.2%) than an improvement (10.6%). One has to keep in mind that subjective perception might differ significantly from objective results, and the DemTect most likely is not sensitive enough to detect minor changes. However, the subjective impression might be an important indicator of changes in cognitive functioning. As a consequence for clinical practice, we would suggest screening the patients reporting a subjective decline in cognitive functioning.

Lower DemTect scores were associated with older age, male gender, and a lower educational level which is in line with previous studies (9). It is well known that the prevalence of cognitive impairment increases with age (31), but this occurs earlier in life in KTx patients than in the general population (7). However, when using logistic regression analysis with the DemTect (dichotomous) as the dependent variable, age did not remain significantly associated. The result supports the allegation that when using the age-adapted DemTect scores for patients older and younger than 60 years, no further adaptions are required to control for age (13).

Concerning gender, female participants showed significantly less often cognitive impairment compared to male participants. This difference remained significant after controlling for other significantly associated variables such as eGFR, hypertension, and educational level and cannot be explained by differences in these variables between genders. It is well known that probably due to hormonal effects, atherosclerosis aggravates slower in women than in men resulting for instance, in a 5-year gap between men and women suffering from stroke (32). Of note, CKD is associated with abnormalities in sex hormones, and KTx has been shown to normalize serum sex hormone concentrations (33, 34). Thus, female sex hormones might exert a protective effect against CNI induced progression of atherosclerosis.

There were significantly more patients with 12 or more years of education in the group without cognitive impairment compared to the other group. A recent review of Lenehan et al. (35) suggests that people with a higher educational level can perform longer at a higher level compared to less educated individuals. Therefore, it takes more time until cognitive impairment becomes apparent. However, it remains unclear if there is a difference regarding the cognitive decline in higher and lower educated individuals (35).

The rate of patients diagnosed with hypertension was significantly higher in the group with cognitive impairment. This finding is in accordance with previous results showing that high blood pressure, especially at middle age, is associated with a higher risk for cognitive impairment (36). Possible mechanisms might be an increased occurrence of atherosclerosis and other cardiovascular comorbidities (36). Due to the design of our study, we have no information on the way hypertension has been treated in the past in our participants. Nevertheless, our results support the association between hypertension and cognitive impairment. From a clinical perspective efficient treatment of hypertension is strongly recommended.

Other medical conditions such as diabetes mellitus and anemia can be associated with cognitive impairment as well (37–39); however, in our study, no differences between the groups were found regarding the rate of renal anemia and diabetes mellitus. One explanation might be that we took the diagnoses from the patient charts without any further information on the current status of the disease. Especially anemia can be treated successfully, leading to a complete remission of associated symptoms. Therefore, the occurrence of the diagnosis might not be automatically associated with the presence of symptoms and possible long-term effects.

In patients with CKD—and not exclusively ESRD—it is well known that cognitive impairment increases in prevalence and severity with declining kidney functioning (7, 40, 41). In our study, participants with cognitive impairment had a significantly lower eGFR compared to those without cognitive impairment. In contrast to the results of Gupta et al. (9), our findings support the hypothesis that mechanisms responsible for cognitive impairment in patients with CKD might also play an essential role in cognitive functioning in KTx patients with reduced eGFR as suggested by Van Sandwijk et al. (8).

Donation-specific variables seem to play a marginal role in cognitive functioning after KTx. While there is evidence that shorter time on dialysis and living kidney donation are associated with a better outcome after transplantation (42), this was not—and in the case of living kidney donation marginally—associated with cognitive functioning in our sample. Also, we found no association between time passed since KTx and cognitive functioning. In conjunction with the results described above, these findings suggest that kidney function might have a stronger association with cognitive functioning compared to donation-specific variables.

It is well known that non-adherence to immunosuppressants is a risk factor for transplant rejection (43). One reason for non-adherent behavior might be cognitive impairment (44, 45). However, we found no difference regarding self-reported adherence between patients with and without cognitive impairment. On the one hand, impaired cognitive functioning can be compensated by social support and practical strategies to improve adherence (45). On the other hand, self-report instruments might not be the perfect tool to assess adherence in patients with cognitive impairment because they might not remember correctly if they have taken their medication.

Anxiety and depression are common mental disorders in the general population and have an even higher prevalence in patients after organ transplantation (46). Depression is known to affect cognitive functioning (47). In our study; however, there was no difference regarding symptoms of depression and anxiety between the patients with and without cognitive impairment. In both groups the mean scores for the subscales “depression” and “anxiety” were clearly below the suggested cut-off of 11 (**Table 2**), indicating low symptom severity in both groups which might explain the lack of association with cognitive functioning.

In a subgroup of our sample, measured weight and height were available. We found a significantly higher rate of obesity (BMI > 30 kg/m^2^) in the group of patients with cognitive impairment in comparison to the group without cognitive impairment. The rising overall prevalence of obesity in the general population is mirrored in the transplant population and prevalence rates of 16% to 20% can be found (48, 49). This finding is in good agreement with other results: In a recent review by Monda et al. (50), a significant association was detected between obesity and cognitive impairment. Additionally, obesity in patients after KTx is associated with a higher risk for adverse events including higher rates of diabetes mellitus, dyslipidemia, and hypertension as well as a higher risk of mortality and graft failure (51). With declining graft function and hypertension being identified as risk factors for cognitive impairment in KTx patients, it becomes evident that obesity influences cognitive functioning at least indirectly. However, the evidence is still lacking if obesity is an independent risk factor for cognitive impairment (50).

Some limitations are worth noting. Due to the cross-sectional design of our study, we can only describe correlates and can make no assumptions regarding cause and effect. Another limitation is the use of a single screening measure of cognitive function (the DemTect) that is unable to detect domain-specific effects compared to comprehensive neuropsychological test batteries. As there are no baseline data available before transplantation, we do not know if there are objective changes in cognitive functioning after transplantation. More detailed information on comorbidities and complications post-treatment, such as infections or acute rejection episodes that may also influence cognitive function were not captured in our study. Finally, we have to take the possibility of a selection bias into account as we cannot be sure that the 619 participating KTx patients are representative of the whole population of KTx patients.

In conclusion, the findings of our study suggest that cognitive impairment affects a significant number of patients after KTx. This is of relevance since cognitive dysfunction has shown to be associated with an elevated risk of graft loss and also often precedes dementia. Furthermore, we were able to identify variables that are associated with cognitive impairment such as male gender, lower educational level, the presence of hypertension, and lower kidney functioning. Since the clinical perception of cognitive impairment by nurses and physicians has shown to be inaccurate in detecting cognitive impairment in KTx patients (52), transplant centers may consider screening for cognitive impairment using objective tests especially in patients with a high-risk profile. Centers should address cognitive decline by implementing management strategies such as cognitive or physical exercise training. However, the evidence for available strategies is still limited, and further research is required in this field. We will continue to evaluate cognitive functioning longitudinally in the participants of our study since cognitive trajectories over a longer period, putative moderators, and mediators of change are still understudied.

## Data Availability Statement

The datasets generated for this study are available on request to the corresponding author.

## Ethics Statement

The studies involving human participants were reviewed and approved by Institutional Ethics Review Board of Hannover Medical School (Number 3464–2017). Written informed consent to participate in this study was provided by the participants’ legal guardian/next of kin. All subjects gave written informed consent in accordance with the Declaration of Helsinki.

## Author Contributions

LP, MS, MZ, and UT designed the KTx360° trial and obtained research funding. MZ, MN, and MS designed this substudy. MN, FK, MB-H, and E-MKTT 3 collected the data. MZ and MN analyzed the data. MN wrote the first draft of this paper. All authors critically revised the manuscript and read and approved the final version. LS was essential in the recruitment process of the study.

## Funding

The study is supported by a grant the Federal Joint Committee of the Federal Republic of Germany under the number 01NVF16009.

## Conflict of Interest

The authors declare that the research was conducted in the absence of any commercial or financial relationships that could be construed as a potential conflict of interest.
